# East–West Disparities in Lung Cancer Screening: Subsolid Nodule Prevalence, Interval Growth, and Decision-Making Analysis

**DOI:** 10.3390/diagnostics16101442

**Published:** 2026-05-08

**Authors:** Yi-Chi Hung, Yun-Ju Wu, Fu-Zong Wu

**Affiliations:** 1 Department of Radiology, Kaohsiung Veterans General Hospital, Kaohsiung 813414, Taiwan; 2 Department of Medical Education and Research, Kaohsiung Veterans General Hospital, Kaohsiung 813414, Taiwan; 3 Laboratory of Tissue-Engineering, Department of Medical Imaging and Radiological Sciences, Central Taiwan University of Science and Technology, Taichung 40601, Taiwan; 4 Institute of Education, National Sun Yat-sen University, 70, Lien-hai Road, Kaohsiung 80424, Taiwan; 5 Faculty of Medicine, School of Medicine, National Yang Ming Chiao Tung University, Taipei 112, Taiwan

**Keywords:** sub-solid nodules, lung cancer screening, decision making

## Abstract

Lung cancer screening has been widely studied, and strong evidence supports its role in reducing mortality among heavy smokers. The 2011 National Lung Screening Trial demonstrated a 20% reduction in lung cancer mortality, with further validation from European trials, such as NELSON and MILD. In 2015, the United States Centers for Medicare & Medicaid Services approved lung cancer screening as a reimbursable service, later expanding the criteria in 2021 to include individuals aged 50–80 years with a ≥20 pack-year smoking history. While screening models such as the Prostate, Lung, Colorectal, and Ovarian (PLCO) have effectively stratified risk among smokers, emerging research on non-smokers remains inconclusive. This review highlights five key issues in lung cancer screening in Eastern and Western countries. First, the screening rates differ significantly between regions owing to variations in healthcare policies and awareness. Second, subsolid nodule (SSN) prevalence varies between Eastern and Western populations, influencing screening strategies. Third, differences in SSN growth thresholds affect clinical decision-making and patient outcomes. Fourth, there are variations in the management of SSNs, particularly in follow-up recommendations and intervention strategies. Fifth, overdiagnosis remains a critical concern, with distinct challenges in each region owing to screening frequency and healthcare infrastructure. Additionally, microsimulation models predict a decline in smoking-related lung cancer but an increase in non-smoking-related cases, emphasizing the need for tailored screening approaches. Addressing these five issues is crucial for optimizing lung cancer screening strategies and balancing early detection with the risk of overdiagnosis.

## 1. Introduction

This literature review mainly discusses the recent evidence and new trends in lung cancer screening. The 2011 National Lung Screening Trial (NLST) confirmed a 20% reduction in lung cancer mortality among heavy smokers [1]. Subsequent studies in Europe and America, such as the Nederlands Leuvens Longkanker Screenings Onderzoek (NELSON) and Multicentric Italian Lung Detection (MILD) trials, further demonstrated that lung cancer screening for heavy smokers can reduce mortality rates [2,3]. Further systemic reviews and meta-analyses have supported the notion that screening heavy smokers, who are at high risk, can reduce the risk of lung cancer mortality (RR = 0.84, 95% CI 0.75–0.93) [4]. Therefore, in 2015, the United States Centers for Medicare and Medicaid Services (CMS) included lung cancer screening as a reimbursable item [5]. In 2021, supported by emerging evidence and considering the overall low screening rates in the United States, the screening threshold was lowered to 50–80 years of age with a smoking history of >20 pack-years [6,7,8].

Currently, there is sufficient evidence supporting lung cancer screening in heavy smokers [9,10]. High-risk screening primarily targets heavy smokers, utilizing the Prostate, Lung, Colorectal, and Ovarian (PLCO) model to classify individual risk levels, which aids in evaluating the potential benefits and drawbacks of lung cancer screening for individuals [11,12]. Ample empirical evidence is available for reference regarding risk prediction models tailored for heavy smokers. In recent years, research has begun to focus on developing risk prediction models for non-smokers [13,14,15,16,17]. However, there is currently no strong evidence supporting this hypothesis.

The NLST recommends annual screenings [1]. However, recent studies, such as the MILD trial, have adopted a biennial screening interval, which has shown benefits [3]. Therefore, considering the policies, economics, and evidence, the adoption of a biennial screening interval may be an inevitable future trend. In 2013, the United States started using Lung-RADS 1.0 for lung cancer screening reports, which was later updated to LUNG-RADS 2022 in 2022 [18,19]. This update further discusses concepts, such as cystic lung cancer and stepwise management for follow-up. The efficacy of screening for heavy smokers has been validated by numerous clinical trials and meta-analyses of lung cancer mortality [10]. However, research on nonsmokers is ongoing in Japan and mainland China, and future results are worth noting [20,21]. The absence of well-controlled randomized clinical trials in never-smoker populations remains a major limitation of the current evidence base. While large-scale studies such as the National Lung Screening Trial and the NELSON Trial have demonstrated the effectiveness of LDCT screening in high-risk heavy smokers, their results are not directly generalizable to never-smokers due to fundamental differences in risk profiles, tumor biology, and environmental exposures.

Additionally, microsimulation model data suggest that smoking-related lung cancer will continue to decrease, while non-smoking-related lung cancer will increase annually from 1980 to 2070 [22]. Therefore, this study aimed to conduct a literature review of five key questions related to lung cancer screening in Eastern and Western countries. In the literature, subsolid nodules (SSNs) are generally classified into part-solid nodules (PSNs) and ground-glass nodules (GGNs). “Pure GGN” is commonly used interchangeably with GGN, referring to non-solid lesions without any solid component. These questions included the prevalence of subsolid nodules (SSNs), screening rates, SSN interval growth threshold, clinical decision-making, and the impact of potential overdiagnosis. This article examines these topics, emphasizing their significance in the field of lung cancer screening.

## 2. Lung Cancer Prevalence Rate

The first issue concerns the lung cancer screening rates in Eastern and Western countries, shown in Table 1. The United States initiated real-world implementation of lung cancer screening using Medicare and Medicaid in 2015 [23]. However, the results and effectiveness of clinical trials and real-world implementations often differ, with various obstacles and limitations that are not observed in clinical research [24,25]. Recent studies have explored the effectiveness of low-dose computed tomography (LDCT) lung cancer screening in various Eastern and Western countries [7,20]. According to the American Lung Association, the overall lung cancer screening rate in the United States is only 5.8%, with significant heterogeneity observed among the different states. California had the lowest rate at 1%, whereas Massachusetts had the highest rate at 16.3% [6,26]. Therefore, increasing screening rates among heavy smokers is an important issue faced in the real world in the United States [27,28]. However, Eastern countries face different challenges. In the Asian Prospective Lung Cancer Screening project, the screening rate among high-risk smokers was 41.9%, whereas that among high-risk nonsmokers was 66.3% [29]. This indicates that the screening rate among high-risk nonsmokers is much higher than that among high-risk smoking populations in the United States. An analysis of lung cancer screening rates in various Asian countries based on literature data revealed that the average screening rate in mainland China ranges from 6 to 31%, whereas in South Korea, it is 23% [6]. Because risk prediction models for high-risk nonsmokers in Asian countries have not been clearly defined, recent studies have found that using LDCT screening in high-risk nonsmokers may lead to overdiagnosis, especially among female nonsmokers [13,30]. Therefore, implementing sex-specific screening and recruitment policies in Asian countries could help avoid overdiagnosis. Applying non-smoking high-risk prediction models to female populations can reduce the potential impact of overdiagnosis, while strengthening screening recruitment rates and advocacy efforts among male populations with heavy smoking can optimize screening benefits and increase male screening willingness [14,31]. Through such comprehensive strategies, the existing challenges of lung cancer screening in Asian countries can be addressed more effectively, thereby improving the success rate of screening programs and societal health benefits.

## 3. Disparity in the Prevalence of SSNs

The second issue examines the disparity in the prevalence of SSNs between Eastern and Western countries. Currently, no original studies have directly compared the prevalence rates of SSNs in lung cancer screening in Eastern and Western countries. In 2024, Wu et al. conducted a prevalence rate meta-analysis to investigate the prevalence of SSNs in Eastern and Western countries. The results showed pooled prevalence rates of 12.6% and 3.6% in Asian and Western countries, respectively [32]. Furthermore, subgroup analysis revealed statistically significant differences in the prevalence rates of SSNs between Eastern and Western countries, indicating that Asian populations may have a significantly higher proportion of SSNs than Western populations, possibly due to environmental and genetic factors (such as air pollution and family history of lung cancer). Given the substantial correlation between persistent SSNs and lung adenocarcinoma pedigrees, conducting large-scale lung cancer screening in Asian populations may significantly increase the likelihood of overdiagnosis [33,34]. Clinical management decisions for the high proportion of persistent SSNs found in Asian non-smoking populations will greatly impact the quality of lung cancer screening. Delayed management may affect patient prognosis, whereas aggressive surgical intervention may significantly increase the risk of overdiagnosis [35]. In the future, it will be crucial to establish lung cancer risk prediction models for non-smoking populations to optimize the efficiency of lung cancer screening in Asia and reduce the occurrence of potential overdiagnosis [13,36].

The third issue was to explore literature reviews focusing on different growth thresholds of SSNs in Eastern and Western countries and their impact on real-world decision-making [35]. Through the literature review, we will focus on the current research on SSN interval growth, particularly emphasizing studies utilizing different growth thresholds, such as 2 mm and 5 mm, volume increase by 25%, and stage shift growth. These findings will help us gain a more comprehensive understanding of the characteristics and differences in SSN growth between Eastern and Western countries, as well as their impact on clinical decision-making under different threshold settings.

Regarding the topic of a 2 mm growth threshold, there is currently a considerable amount of research literature (up to 24 articles in Table 2) [34,37,38,39,40,41,42,43,44,45,46,47,48,49,50,51,52,53,54,55,56,57,58,59]. However, the majority of relevant studies come from Asian countries, with only one study from Italy investigating issues related to the 2 mm growth threshold. Synthesizing these literature results shows that during the follow-up period, the rate of PSNs reaching the 2 mm growth threshold was higher than that of ground-glass nodules (GGNs), highlighting the heterogeneity in the growth trends of SSNs. There are currently 7 studies exploring interval nodule growth by volume measurement, with the majority of relevant studies (six) coming from Asian countries, and only one sporadic article from the Netherlands using volume changes to investigate the natural growth history of SSNs in Table 3 [60,61,62,63,64,65,66]. Among these, seven studies addressed interval nodular growth by clearly defining the volume growth interval threshold. According to the review results of this study, Eastern countries have relatively more literature on growth thresholds, and the prevalence of SSNs in Asian countries is higher than in Western countries. This indicates the existence of biological differences in the natural growth history of SSNs between Eastern and Western countries, leading to differences in the prevalence of SSNs and natural threshold growth history.

Currently, relevant clinical management guidelines for SSNs were derived from Western countries [19,67,68]. For example, Fleischer guidelines define 2 mm as the interval growth threshold for SSNs [67]. The Lung-RADS guidelines define 1.5 mm as the interval growth threshold [19]. The BTS guidelines adopt a 25% volume increase as the interval growth threshold [69]. Different definitions of growth thresholds may result in inconsistent clinical decisions and management [35]. Many literature studies have shown that SSNs in Asia exhibit heterogeneous growth rates [70,71]. In 2018, Tang et al. used three different growth thresholds to explore the natural interval growth of SSNs in Asia [46]. Regarding the 2 mm growth threshold, it typically takes 7 years for GGNs to reach the threshold, while PSNs require 3 years. Within a 5-year average follow-up period, 35.5% of ground-glass nodules and 67.3% of PSNs grew. Regarding the 5 mm growth threshold, it typically takes 9 years for GGNs to reach the threshold, while PSNs require 3 years. Within a 5-year average follow-up period, 10.6% of GGNs and 67.3% of PSNs grew. It typically takes 12 years for GGNs to reach the stage shift growth threshold, whereas PSNs require 9 years. Within a 5-year average follow-up period, 0% of GGNs and 24.2% of PSNs will grow. This study demonstrated significant differences in natural progression among distinct nodule types, such as GGNs and PSNs, providing information for personalized clinical decision-making in the development of subsequent follow-up guidelines and management strategies for Asian populations with SSNs.

Kobayashi et al. explored the natural growth history of SSNs over a long period of >10 years [37,72]. Within a 5-year follow-up period, 41% of patients reached a growth threshold of 2 mm. The proportion of individuals reaching an interval growth of 2 mm after 10 years decreased to 13%, indicating heterogeneous growth behavior. After 10 years of follow-up, there was increased stability in the SSNs, with a lower tendency for growth.

Therefore, the application of personalized nodule risk prediction models to determine the optimal clinical nodule management strategy is important for lung cancer screening in Asians. Further, determining whether SSNs are lung cancers during Asian lung cancer screening is not the most important clinical issue. Instead, it is used to diagnose whether SSNs are invasive lung cancers [35,73,74]. Predictive models can be used to predict invasive lung adenocarcinoma and facilitate early surgical management, thereby reducing the risk of delayed diagnosis [75,76]. For early-stage lesions, an active surveillance strategy is adopted to avoid overdiagnosis and false positives, thereby improving the lung cancer screening quality [77].

## 4. Clinical Management of SSNs Perspectives

The fourth issue was to explore the differences in the clinical management of SSNs between Eastern and Western countries. In recent years, evidence-based guidelines on surgical approaches for early-stage lung cancer, such as those from studies by JCOG0802/0804/1211, have gradually reached a consensus [78,79,80]. According to the clinical guidelines in Europe and the United States, pure GGNs should be actively monitored for long periods. If patients strongly desire surgical treatment after shared decision making, wedge resection is prioritized [19,77]. For some SSNs, if the consolidation-to-tumor ratio (CTR) is <0.5, segmentectomy is currently recommended as the preferred surgical treatment [78,79,80]. However, for a CTR > 0.5, lobectomy is recommended for central lesions, whereas segmentectomy is recommended for peripheral lesions.

In 2018, Silva et al. compared long-term survival rates between an SSN-derived lung cancer group and a group with no history of cancer using an active surveillance strategy [77]. This study found no significant differences in the survival curves between the two groups. This conclusion suggests that the long-term active surveillance of SSNs detected through screening is a safe strategy to reduce the risk of overtreatment. Therefore, Western countries have mainly adopted active surveillance strategies for pure GGNs, based on the current clinical standards. Guidelines for SSNs are primarily derived from Western clinical standards. For instance, the Fleischer guidelines define 2 mm as the interval growth threshold for SSNs, whereas the Lung-RADS uses 1.5 mm, and the BTS guidelines use a 25% increase in volume as the threshold. Thus, differences in growth thresholds may lead to varying clinical judgments and management approaches [35]. Considering the high prevalence of SSNs in non-smoking populations in Asian countries, as well as the general concern and anxiety regarding non-smoking-related lung cancer in Asian populations, Asians tend to prefer surgical treatment, which differs from the cultural practices in Western countries. Wu et al. conducted a retrospective analysis of 1978 individuals using a modified Lung-RADS [81]. In the study population, 246 individuals had SSNs. In the GGN group, 167 patients had a surgical treatment rate of 6.6% during the study period. In the PSN group, there were 79 individuals, with a surgical treatment rate of 28.8% during the study period [81]. Thus, based on 246 individuals with SSNs in an Asian lung cancer screening cohort, the overall surgical rate was 12.19%. This indicates that the influence of the high prevalence of SSNs in Asian countries and the cultural awareness of health security in Asia leads to significantly higher surgical rates and number of surgeries in the population with SSNs than in Western countries [82].

Chang et al. found a significant positive correlation between invasive lung cancer and the first-degree relative grade of lung cancer in Taiwan’s first non-smoking lung cancer prospective clinical trial [Taiwan Lung Cancer Screening for Never-Smoker Trial, TALENT] [83]. However, among the patients diagnosed with lung cancer during the study, 19.2% were diagnosed with adenocarcinoma in situ. Since 2011, evidence suggests that lung cancer screening in non-smoking Asian populations may be associated with potential overdiagnosis, which can subsequently lead to overdiagnosis and overtreatment [30,84,85]. Therefore, predicting whether subsolid nodules discovered during baseline checks will increase in the future is an important issue in lung cancer screening for non-smoking populations in Asia [86].

**Figure 1:**
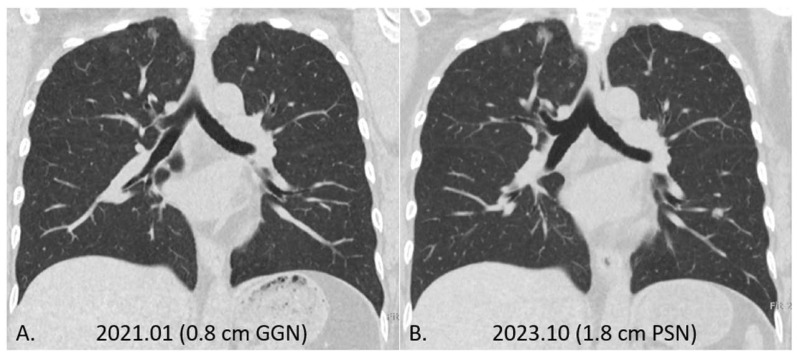
A 50-year-old woman with a family history of lung cancer underwent a baseline CT scan of the lungs in January 2021, which identified a 0.8 cm pure ground-glass nodule in the right upper lobe (**A**). An active surveillance approach was selected for follow-up. Over the next two years and nine months, the nodule exhibited interval growth, increasing to 1.8 cm (**B**). Surgical resection later confirmed the diagnosis of invasive lung adenocarcinoma. This case underscores the critical need for careful monitoring and comprehensive assessment of subsolid nodules, particularly in non-smokers with a familial predisposition to lung cancer, who face an elevated risk of progression to invasive malignancy. Identifying high-risk lesions necessitates a multifaceted evaluation incorporating clinical, imaging, and genetic factors.

The risk of invasive lung cancer is significantly higher in nonsmokers, especially those with a family history of lung cancer [83]. Particularly for individuals with a family history of lung cancer, the likelihood of future growth of SSNs is relatively high. For example, a 50-year-old woman underwent a baseline computed tomography (CT) scan of the lungs in January 2021, revealing a 0.8 cm pure GGN in the right upper lung, shown in Figure 1. We decided to adopt an active surveillance strategy for follow-up. After 2 years and 9 months of follow-up, the nodule showed an interval growth trend, growing to 1.8 cm, and was eventually surgically confirmed to be an invasive lung adenocarcinoma. This case emphasizes the need for careful consideration and close monitoring of changes in SSNs, particularly in nonsmokers with a family history, as they are at a higher risk of developing invasive lung cancer.

Wu et al. predicted interval growth changes in SSNs using combined clinical imaging features [86]. The results showed that a model integrating clinical and radiological features could predict nodule growth changes during staging based on five parameters. Using a line chart to predict the growth status during staging further enhances the practicality of actual clinical applications. The diagnostic performance of this model reached a high area under the receiver operating characteristic curve (AUC) of 0.869. Therefore, clinicians can make appropriate clinical decisions based on the results predicted by line charts and provide individualized surgical recommendations, thereby achieving personalized precision medicine.

## 5. Overdiagnosis Issue

The fifth issue is the comparison of overdiagnosis in Eastern and Western countries. Overdiagnosis is an inevitable outcome of screening programs [87]. Overdiagnosis may lead to downstream overmanagement, but these represent conceptually different processes. This review addresses the extent and impact of potential overdiagnosis of lung cancer screening in Eastern and Western countries. Vachani et al. conducted a study across four healthcare systems in the United States to evaluate the effectiveness of LDCT lung screening [88]. Their study found an increase in the detection of early-stage lung cancer and a decrease in the incidence of late-stage lung cancer following the implementation of lung cancer screening. Importantly, there was no significant increase in the overall lung cancer detection rate, indicating a relatively low overdiagnostic burden. This highlights the effectiveness of LDCT as a screening tool for heavy smokers in the United States, as it aids in early cancer diagnosis and treatment while minimizing the risk of advanced disease and the negative impact of overdiagnosis.

However, in Asian countries, where there is a high prevalence of non-smoking-related lung cancer and a widespread fear of unknown causes, screening programs typically target both smoking and non-smoking populations [13]. However, recent lung cancer statistics from mainland China, South Korea, and Taiwan have shown a rapid increase in the proportion of early-stage lung cancer, particularly among women and younger populations, following large-scale LDCT interventions [self-paid or budget support] [30,84,85]. This suggests that the widespread application of LDCT screening in Asian populations may lead to overdiagnosis, especially among non-smoking and young female populations. However, chest radiography is the primary screening tool for lung cancer in Japan [89]. Nonetheless, recent lung cancer statistics from mainland China, South Korea, and Taiwan indicate a rapid increase in the proportion of early-stage lung cancers following large-scale LDCT interventions, especially among women and younger populations. This indicates that the widespread adoption of low-dose chest CT scans in Asian populations may lead to potential overdiagnosis, especially among non-smoking and young female populations [30,84,85]. However, in Japan, where chest radiography is the primary screening tool for lung cancer, Nguyen et al. suggested adopting large-scale chest X-ray screening for lung cancer and observed some screening effects, including the discovery of more early-stage and late-stage lung cancer [89]. This may imply the moderate effectiveness of nationwide chest X-ray screening for lung cancer screening, with no apparent potential for overdiagnosis.

Chang et al. conducted a prospective clinical trial on lung cancer in non-smoking populations in Taiwan and found a correlation between invasive lung cancer and first-degree relatives of patients with lung cancer [83]. However, among the patients with lung cancer diagnosed during the study, a significantly high proportion of 19.2% were diagnosed with adenocarcinoma in situ. These results indicate that lung cancer screening in non-smoking populations in Asian countries may lead to potential overtreatment and overdiagnosis.

## 6. Conclusions

Owing to the heterogeneity of lung adenocarcinoma lineage growth trends in Asian non-smoking countries, applying highly sensitive LDCT screening tools may lead to a change only in the tip of the iceberg [35]. Clinically asymptomatic indolent lung cancers (hidden under the iceberg, usually presenting as GGNs) are continuously diagnosed during screening because of the security culture preference for minimally invasive thoracoscopic sublobar surgery rather than an active surveillance strategy in Eastern countries, resulting in potential overdiagnosis [83,84].

**Figure 2:**
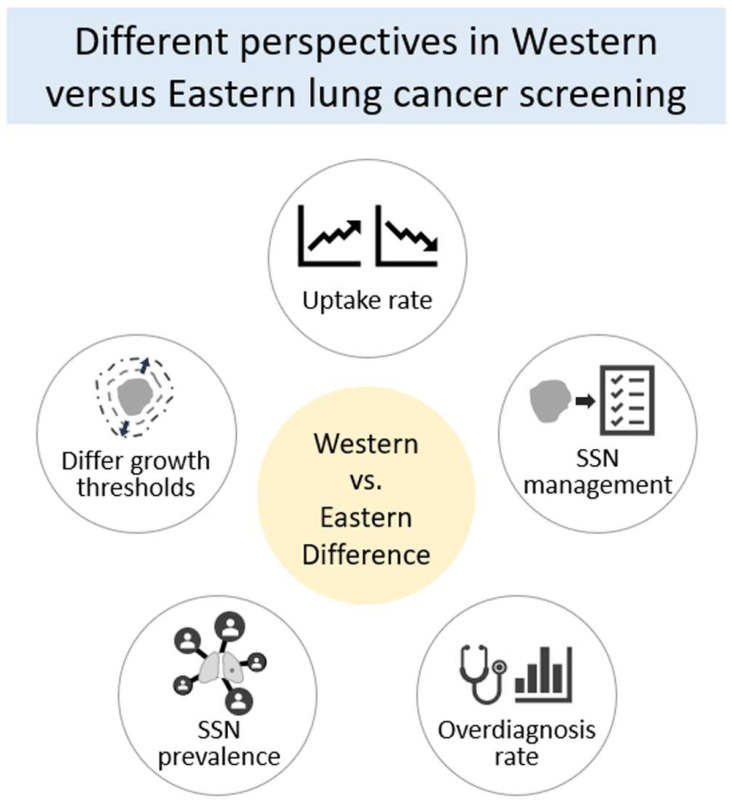
Key differences in Western and Eastern lung cancer screening approaches, highlighting differences in uptake rate, SSN management, overdiagnosis rate, SSN prevalence, and growth thresholds. These factors influence regional strategies for detecting and managing subsolid nodules and lung cancer risks.

Therefore, overdiagnosis is an inevitable phenomenon in lung cancer screening, although it is more pronounced in Asian lung cancer screening because of factors such as the prevalence of GGNs and Eastern preference for thoracoscopic sublobar surgery [16]. Overdiagnosis is an inevitable by-product of lung cancer screening. However, we can mitigate the occurrence of overdiagnosis in Asian lung cancer screening implementation and maintain the ideal effectiveness of lung cancer screening through the following strategies in Figure 2:

The first are government policies that allow the entire population to understand the indications for lung cancer screening, high-risk populations, and potential benefits and harms of individual screening through shared decision-making (SDM) strategies [90]. Therefore, targeting high-risk populations for lung cancer screening will enhance the benefits of lung cancer screening and reduce the harms, such as overdiagnosis caused by lung cancer screening. The second is the adoption of non-smoking lung cancer risk prediction models to select appropriate individuals for screening [14]. The use of non-smoking lung cancer risk prediction models can optimize the risk stratification of individuals for non-smoking lung cancer screening and may more accurately select non-smoking individuals at a high-risk of lung cancer who should undergo lung cancer screening, thereby reducing the significant potential for overdiagnosis caused by indiscriminate non-smoking lung cancer screening. Third, sex-specific strategies should be developed to optimize the screening efficiency [13]. As male smokers generally have lower health literacy and are less willing to actively participate in lung cancer screening, promoting the screening rate of high-risk male smoking populations is an important future policy direction. Non-smoking female populations require non-smoking lung cancer risk prediction models to help identify non-smoking populations that meet the true high-risk threshold, and specific strategies can reduce the potential overdiagnosis caused by large-scale non-smoking population screening. The fourth objective is to set appropriate SSN interval growth thresholds for Asian populations. Owing to the heterogeneous growth rates of the lung adenocarcinoma lineage expressed by SSNs, setting appropriate nodule growth thresholds suitable for Asian populations may optimize clinical decision-making in lung cancer screening and further avoid overdiagnosis and delayed diagnosis [13,35]. The fifth point is the use of interdisciplinary team cooperation to assess clinical decision-making and surgical interventions, which can comprehensively evaluate and optimize the quality of the entire lung cancer screening process [91]. Through these strategies, we can mitigate the problem of overdiagnosis in Asian lung cancer screening implementation, while ensuring the effectiveness and safety of screening in Table 4.

## Figures and Tables

**Table 1:** Literature review on uptake rate of lung cancer screening in Eastern and Western countries.

Region	Country	Lung Cancer Screening Rate	High-Risk Groups	Challenges Identified	Key Findings
Western	United States	5.8% (overall); 1% (California); 16.3% (Massachusetts)	Heavy smokers	Low screening uptake, significant heterogeneity among states	- Screening rates among heavy smokers are low; significant variation in implementation and access by state- - LDCT is the primary screening tool
Eastern	China	6% to 31%	Mixed (smokers and non-smokers)	Lack of standardized risk prediction models for high-risk non-smokers	- Variability in screening rates; potential for overdiagnosis in non-smokers, especially females. - LDCT is the primary screening tool
Eastern	South Korea	23%	Mixed	Need for tailored strategies to optimize screening rates	- Moderate screening uptake; higher than the US but faces challenges in risk model application for non-smokers - LDCT is the primary screening tool.
Eastern	Japan	53.4% (Men) 45.6% (Women)	Men and women aged 40–79 years, eligible for screening	- Low public awareness - Psychological barriers	- Screening rate improved over time - No financial/healthcare barriers - Psychological factors affect participation - Chest radiography is the primary screening tool

**Table 2:** Pulmonary subsolid nodules 2 mm interval growth analysis across 24 studies.

Author	Year	Country	Age (Years)	Nodular Size (mm)	Number of Cases	Smoking Habit	Number of Nodules	Nodular Growth Rate, Individual (%)	Follow-Up Period (Year/Month)
Hyun Woo Lee [37]	2019	South Korea	52	4.7	160	49.4% (79/160)	208	15.6% (25/160)	11.33 years
Miyako Hiramatsu [38]	2008	Japan	62	8.3	125	32.8% (41/125)	125	21% (26/125)	2.87 years
Silva Mario [39]	2012	Italy	59.7	6.97	56	N/A	76	N/A	4.18 years
Boksoon Chang [40]	2013	South Korea	53	5.5	89	66.3% (59/89)	122	13.5% (12/89)	4.9 years
Haruhisa Matsuguma [41]	2013	Japan	60	11.27	171	32.7% (56/171)	174	23.9% (41/171)	2.42 years
Sei Won Lee [42]	2013	South Korea	61	7.8	114	44.7% (51/114)	175	39.5% (45/114)	3.75 years
Yoshihisa Kobayashi [43]	2013	Japan	61	9.5	61	31.1% (19/61)	108	N/A	4.2 years
Takashi Eguchi [44]	2014	Japan	64.5	7.4	124	20.2% (25/124)	124	51.6% (64/124)	4.75 years
Yoshihisa Kobayashi [45]	2014	Japan	60	≤30	67	32.8% (22/67)	120	N/A	4.2 years
En-Kuei Tang [46]	2018	Taiwan	60.58	9.98	128	N/A	128	46.9% (60/128)	3.57 years
Ryutaro Kakinuma [47]	2016	Japan	62	≤30	795	39.9% (317/795)	1229	N/A	4.3 years
Yangbo Qiu [48]	2018	China	61	N/A	143	N/A	143	34.9% (50/143)	1.16 years
Hai Xu [49]	2019	China	52	4.9	693	N/A	693	7.5% (52/693)	2.4 years
Chen Gao [50]	2020	China	57.4	8.216	85	N/A	110	N/A	>2 years
Jong Hyuk Lee [51]	2016	South Korea	57.88	5-30	213	32.86% (70/213)	213	N/A	849 days
Taichun Qiu [52]	2020	China	59.525	8.45	80	N/A	80	N/A	3.79 years
Zhe Shi [53]	2019	China	61	9.75	59	20%	59	N/A	52 months
Sato [54]	2017	Japan	65.5	12.2	187	62%	187	33.2% (62/187)	44.4 months
Masaya [55]	2014	Japan	70.8	11.4	53	33.3% (21/63)	63	N/A	26.1 month
Wang [56]	2017	China	59	<30	169	N/A	203	N/A	24 months
Yeon wook kim [34]	2021	South Korea	52.2	6.89	4545	N/A	3643	N/A	47.1 months
So Hyeon Bak [57]	2016	South Korea	58.9	11.7	49	40.8% (20/49)	54	N/A	24.2 months
Jaeyoung Cho [58]	2016	South Korea	56	5	218	38.5% (84/218)	453	6.4% (14/218)	77.5 months
Takahashi [59]	2012	Japan	62.6	8.14	111	N/A	150	N/A	66 months

Abbreviation: N/A, not applicable.

**Table 3:** Analysis of pulmonary subsolid nodule growth volume insights from 7 studies.

Author	Year	Country	Age (Years)	Nodular Size (mm)	Number of Cases	Smoking Habit	Number of Nodules	Nodular Growth Rate, Individual (%)	Follow-Up Period (Year/Month)	Threshold Definition
Yong Sub Song [60]	2014	South Korea	58	10.15	97	N/A	97	29.9% (29/97)	1.73 years	Volume growth 30%
Lin-Lin Qi [61]	2020	China	54.3	8.7	110	N/A	110	47.3% (52/110)	4.06 years	Volume growth 20%
Xianqun Xu [62]	2017	China	52.1	15.513	176	N/A	69	N/A	N/A	Volume growth 20% (>10 mm); Volume growth 30% (5–9 mm)
Kyung Eun Shin [63]	2014	South Korea	55	7	70	N/A	70	N/A	61 months	Volume growth 25%
Scholten [64]	2015	Netherlands	62	11.75	234	52.8% (57/234)	101	N/A	95 months	Mass volume growth 30%
Yifan He [65]	2023	China	65	6.4	154	5.3%	154	N/A	66 months	Volume growth 25%
Shigeki Sawada [66]	2009	Japan	64	10	113	N/A	113	23.9% (27/113)	0.76 years	Volume growth

Abbreviation: N/A, not applicable.

**Table 4:** Five strategies to reduce overdiagnosis of lung cancer screening in Asia.

Strategy	Description	Expected Outcome
Government policies and SDM strategies	Educate the population on lung cancer screening indications, high-risk populations, and benefits/harms. Use Shared Decision-Making (SDM).	Enhanced understanding of screening benefits and harms; targeted screening for high-risk groups to reduce overdiagnosis.
Non-smoking lung cancer risk models	Adopt models to optimize risk stratification for non-smoking populations.	Accurate identification of high-risk non-smokers; reduced overdiagnosis in indiscriminate non-smoker screenings.
Gender-specific strategies	Develop policies to improve screening rates in male smokers and optimize non-smoker female screening using risk models.	Increased male smoker participation; minimized overdiagnosis in non-smoking females through precise risk stratification.
SSN interval growth thresholds	Set nodule growth thresholds suitable for Asian populations based on the heterogeneous growth of lung adenocarcinomas.	Optimized clinical decision-making; reduced overdiagnosis and delayed diagnosis.
Interdisciplinary team collaboration	Use multi-disciplinary teams for clinical decision-making and surgical interventions.	Improved screening process quality; comprehensive assessment and optimization of clinical and surgical outcomes.

## Data Availability

No new data were created or analyzed in this study. Data sharing is not applicable to this article.
